# Qualitative impact assessment of an educational workshop on primary care practitioner attitudes to NICE HIV testing guidelines

**DOI:** 10.3399/bjgpopen18X101433

**Published:** 2018-04-07

**Authors:** Rosalie L Allison, Ellie J Ricketts, Thomas Hartney, Anthony Nardone, Katy Town, Claire Rugman, Kate Folkard, J Kevin Dunbar, Cliodna AM McNulty

**Affiliations:** 1 Research Assistant, Primary Care Unit, Department of Microbiology, Public Health England, Gloucester, UK; 2 Cancer Support Specialist, Oncology Department, Derriford Hospital, Plymouth, UK; 3 PhD student, National Institute for Health Research, Health Protection Research Unit in Blood Borne and Sexually Transmitted Infections, University College London, London, UK; 4 Consultant Scientist (Sexual Health Promotion), HIV/STI Department, Centre for Infectious Disease Control and Surveillance, Public Health England, London, UK; 5 Senior HIV/STI Surveillance Scientist, HIV/STI Department, Centre for Infectious Disease Control and Surveillance, Public Health England, London, UK; 6 Formerly Primary Care Unit, Department of Microbiology, Public Health England, Gloucester, UK; 7 National Chlamydia Screening Programme Manager, HIV/STI Department, Centre for Infectious Disease Control and Surveillance, Public Health England, London, UK; 8 Director, National Chlamydia Screening Programme, HIV/STI Department, Centre for Infectious Disease Control and Surveillance, Public Health England, London, UK; 9 Unit Lead, Primary Care Unit, Department of Microbiology, Public Health England, Gloucester, UK

**Keywords:** General practice, HIV, Qualitative research, condoms, contraception, chlamydia

## Abstract

**Background:**

In 2013, Public Health England piloted the ‘3Cs (chlamydia, contraception, condoms) and HIV (human immunodeficiency virus)’ educational intervention in 460 GP surgeries. The educational HIV workshop aimed to improve the ability and confidence of staff to offer HIV testing in line with national guidelines.

**Aim:**

To qualitatively assess the impact of an educational workshop on GP staff’s attitudes to NICE HIV testing guidelines.

**Design & setting:**

Qualitative interviews with GP staff across England before and after an educational HIV workshop.

**Method:**

Thirty-two GP staff (15 before and 17 after educational HIV workshop) participated in interviews exploring their views and current practice of HIV testing. Interview transcripts were thematically analysed and examined, using the components of the theory of planned behaviour (TPB) and normalisation process theory (NPT) as a framework.

**Results:**

GPs reported that the educational HIV workshop resulted in increased knowledge of, and confidence to offer, HIV tests based on indicator conditions. However, overall participants felt they needed additional HIV training around clinical care pathways for offering tests, giving positive HIV results, and current treatments and outcomes. Participants did not see a place for point-of-care testing in general practice.

**Conclusion:**

Implementation of national HIV guidelines will require multiple educational sessions, especially to implement testing guidelines for indicator conditions in areas of low HIV prevalence. Additional role-play or discussions around scripts suggesting how to offer an HIV test may improve participants’ confidence and facilitate increased testing. Healthcare assistants (HCAs) may need specific training to ensure that they are skilled in offering HIV testing within new patient checks.

## How this fits in

Previous research has highlighted missed opportunities for HIV testing in general practice. National Institute for Health and Care Excellence (NICE) HIV guidelines include encouraging GP staff to routinely offer HIV testing to patients that have symptoms that may indicate HIV or HIV is part of the differential diagnosis (indicator conditions), and to all new patients that register at general practices in areas of high HIV prevalence (>2 diagnoses per 1000 people). The research shows that an educational workshop covering the NICE HIV testing guidelines, with clear indications for testing and how to test, was welcomed by GP staff, and gave them increased knowledge and awareness of when to offer an HIV test in the primary care setting. However, additional support and training is needed in order to help participants feel more confident regarding routinely offering HIV tests. In combination with the workshops (available at http://stitraining.eu/catte-resources/), additional role-play or discussion around scripts used to offer an HIV test would be useful in changing attitudes and helping to implement NICE HIV testing guidelines.

## Introduction

In 2015, Public Health England (PHE) estimated that 101 200 people were living with HIV in the UK, of whom 13% (13 500) were unaware of their infection.^1^ The majority of new infections are transmitted from those who are undiagnosed,^2^ and late diagnosis of HIV is associated with increased morbidity.^3,4^ In 2011, NICE produced guidelines for HIV testing^5,6^ aiming to expand testing in non-genitourinary (GU) medicine settings, including general practice staff.

**Figure 1.**
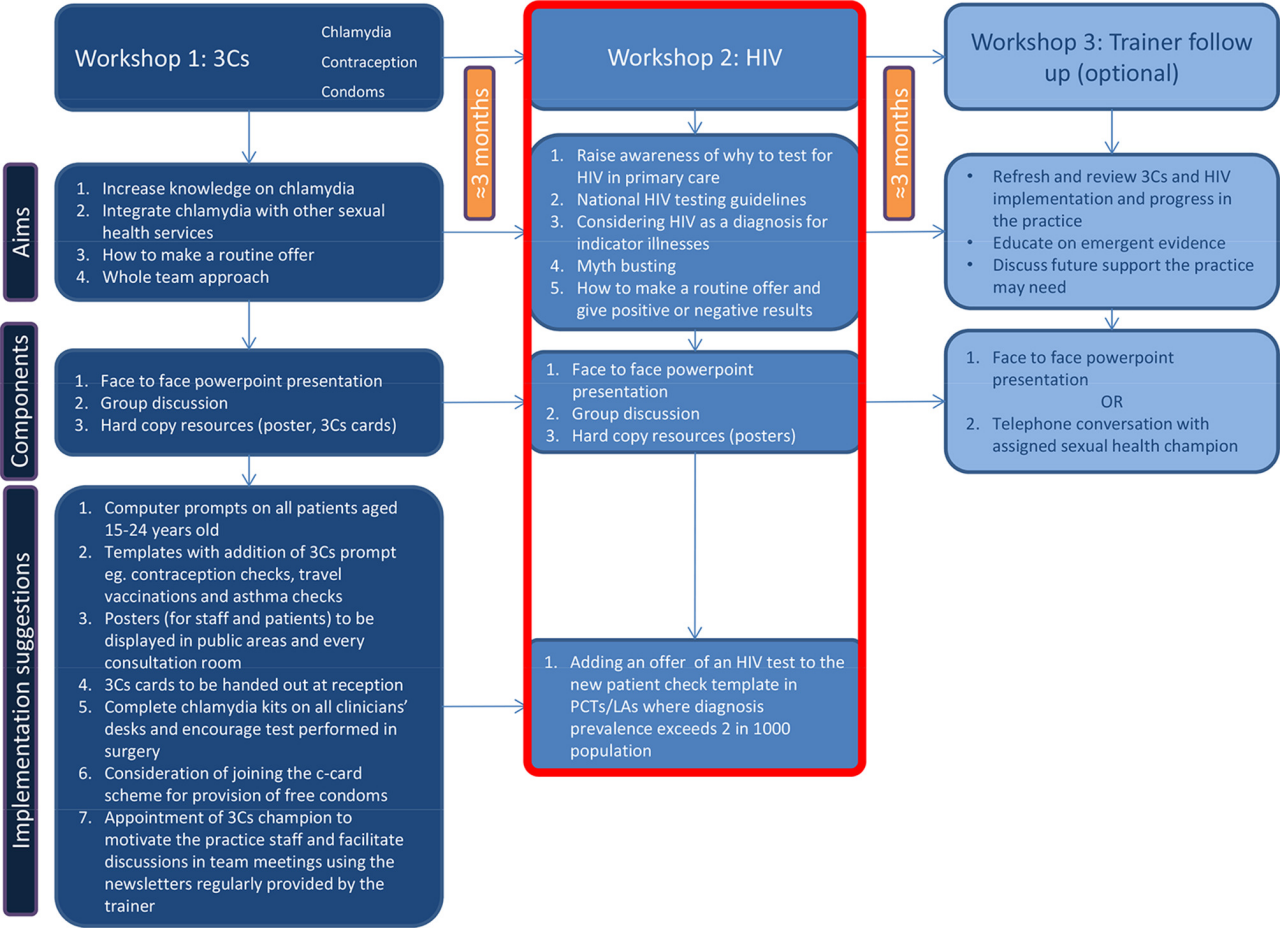
The 3Cs (chlamydia, contraception, condoms) and HIV (human immunodeficiency virus) intervention with the components that aim to increase HIV testing highlighted in red.

In 2013, PHE piloted a multifaceted intervention called the ‘3Cs and HIV’ programme for general practices,^7–9^ aiming to integrate chlamydia screening with other sexual health and reproductive services, and promoting the offer of HIV testing. The 3Cs and HIV intervention, based on the TPB,^10,11^ consisted of two educational workshops, delivered by trained local sexual health staff, and an optional follow-up with the trainer (see Figure 1 for details). The first workshop focused on the routine offer of 3Cs. The second workshop focused on facilitating HIV testing, and encouraged GP staff to offer HIV testing in line with the 2011 NICE guidelines;^5,6^ that is, to patients with HIV indicator condition,^12^ and to all newly registered patients in areas of high HIV prevalence (>2 diagnoses per 1000 people).

Since completion of the intervention, NICE have updated their guidelines on HIV testing,^13^ by expanding them to include:

1. offering a less invasive form of specimen collection, such as mouth swab or finger-prick if a venous blood sample is declined; and
2. incorporating the routine offer of an HIV test into every consultation, for areas with extremely high HIV prevalence (>5 per 1000 people).

This qualitative study aims to assess the impact of an educational workshop on GP staff’s attitudes to 2011 NICE HIV testing guidelines. The results will be used to inform implementation of the 2016 NICE HIV testing guidelines in a GP setting.

## Method

### Sampling and recruitment for qualitative interview

The researchers aimed to recruit GP staff from rural and urban areas with a range of HIV prevalence. Chlamydia screening rates^14^ were used as the indicator for sexual health provision in surgeries; HIV prevalence was recorded and linked to each practice for future reference. Data saturation^15^ was the determinant for the sampling cut-off point.

### Pre-intervention

GP surgeries in three socioeconomically different areas were stratified by 2012 chlamydia screening rate, and the highest testing tertile approached in random order. Purposive sampling^16^ was then used to match practices of low chlamydia testing rates that were closest geographically to the practices already recruited, checking for a range of HIV prevalence. Interviews were carried out face-to-face at GP surgeries.

### Post-intervention

Practices that had participated in the 3Cs and HIV intervention were stratified by chlamydia screening rate from January 2013 until September 2014^7^ and, initially, contacted in a random order. Purposive sampling^16^ was later used to recruit further practices with a range of HIV prevalence. Practice managers were contacted, and telephone interviews with GP staff who had attended the educational workshop were arranged. Because the interview and discussion can be seen to influence attitudes and behaviours around HIV through reflection about current practice, different participants were interviewed before and after the workshop.

### Interview procedure

GP staff provided written, informed consent to participate for audiorecording and for anonymised quotes to be published. Interviews followed a semi-structured interview schedule^17^ (further information available from the authors on request), developed using TPB^11^ and NPT,^18^ and were conducted by practiced interviewers, using probing as opposed to leading questions. GP staff were offered financial incentives to participate.

### Data analysis

Interviews were recorded, transcribed verbatim, anonymised, and imported to NVivo (version 10). Initial thematic analysis^19^ was ongoing and iterative, and further framework^20^ analysis occurred after data collection. Two researchers independently coded categories and themes. The lead researcher coding all transcripts was not involved in intervention development or delivery. A second researcher coding a third of the transcripts was involved in intervention development as part of the larger team. Minor discrepancies over coding language were resolved through discussion, and transcripts revisited to ensure coding consistency.

Initial themes and sub-themes were discussed with the whole study group. From this, descriptive accounts were developed, with illustrative quotes, using the components of TPB and NPT as a framework.^11,18,21^ TPB was used to explore and understand the behaviour, and NPT was chosen to assess implementation of the intervention. The qualitative data was aligned with the components of these frameworks.

## Results

Before the educational HIV workshop, data saturation^15^ was reached after 15 interviews with GP staff: 6 from areas of low HIV prevalence (3 GPs, 3 nurses); and 9 from areas of high HIV prevalence (3 GPs, 6 nurses). After the educational HIV workshop, data saturation was reached after 17 interviews: 14 from areas of low HIV prevalence (7 GPs, 7 nurses); and 3 from areas of high HIV prevalence (1 GP, 2 nurses) (further information available from the authors on request).

The themes that emerged from the interviews are outlined below, presented using the structure of the TPB^10^ and NPT.^18^

### Attitudes towards routinely offering HIV testing, in line with NICE guidance (TPB) (Box 1)

**Box 1.** Quotes on attitudes towards routinely offering HIV testing in-line with NICE guidance (TPB)

'Well, if I explained to them why I was doing it then I’d be very happy to do it.' (GP KTGP30J, pre-intervention, low prevalence) ' ... we don’t actually do new patients health checks on everyone, we don’t have the time or resources.' (Nurse KTPN15K, pre-intervention, high prevalence) 'If we [send] the blood test off, we’re paying for that blood test, um, and that’s why we don’t routinely do it. As far as I know, we pay for that.' (Nurse ER95a, pre-intervention, high prevalence)

Before the educational HIV workshop, no participants were aware of any current practice policies around offering a routine HIV test to newly registered patients. When discussing feasibility of implementing this policy, many participants expressed concerns that not all new patients are offered a check, and there would be money, time, or logistical costs of routinely offering HIV testing. Some nurses suggested that offering an HIV test to newly registered patients would be a good way to offer HIV routinely.

### Subjective norm: the perceived social pressure to offer or not to routinely offer HIV testing, in line with NICE guidance (TPB) (Box 2)

**Box 2.** Quotes on subjective norm: the perceived social pressure to offer or not to routinely offer HIV testing, in line with NICE guidance (TPB)

**'So, on what basis do you think that staff in general practices should offer or recommend an HIV test to their patients?'** 'A lot of the time here, it’s on patient request. Um, if there’s needle or stick injury for staff and, you know, any patients that think they’ve come into contact with, or at risk of. Um, I think it should be more widely tested, but again, it’s done to cost and practicalities.' **'What’s the practicality bit for you?'** ' ... well doesn’t it take a long, long time to get it back? And it’s very costly, um, is that my perception.' (Nurse ER95B, pre-intervention, high prevalence) '[Discussing when they would offer an HIV test] We were screening opportunistically as well for people that were coming in for the sexual health checks um, I think we still need to do better. I think, at the moment, when the surgery thinks sexual health checks they think swabs, for women, and they’re not thinking what we’re doing for men, and thinking that, maybe, we should be routinely offering to anyone ... I think we definitely need to do more HIV side, yeah.' (Nurse KTPN17F, pre-intervention, high prevalence) **'How do you think the patients feel about being recommended an HIV test?'** 'I still think there’s a lot that would be probably quite shocked that we were recommending that.' (Nurse YORA1, post-intervention, low prevalence) **'Do you know what the response is with that? Do people say yes, no?'** 'Certainly the ones I see in consultation, 99% say "yes, have the bloods done for my testing".' (GP YORA4, post-intervention, low prevalence) 'I do, I suppose I would offer it differently to the GP. So I’d offer it as part of the sexual health screening, so if someone’s having a smear done and I say, "would you be interested in a sexual health screen?" then we’d talk about the differences of swabs or the blood borne viruses. It’s interesting that a lot will want chlamydia check but aren’t interested in any of the blood borne virus.' (Nurse KTPN15K, pre-intervention) 'You’ve got the patient in, you test them and wait 20 minutes for the result, and then after the result, you’ve got to chat to them about the results. That’s not just 20 minutes, that time is a lot, lot longer … Doctors get 7–﻿10 minutes per patient and we get 15 minutes, so I don’t think our appointments can accommodate.' (Nurse KTPN97A, pre-intervention, low prevalence) '[Discussing POCTs] If was accurate, which they’re supposed to be, aren’t they? ... I suppose my concern would be that, that the back-up, you know, there was availability, somebody from GU to talk to them and follow them up fairly swiftly … Doing that on somebody that’s high risk, that’s the problem with these things, isn’t it, they’re all fine and dandy when they’re all negative, but if you’ve got a, yeah, a positive in the middle of a morning surgery, you haven’t got an hour to sit with them and discuss the ins and outs and all the rest of it. So that, that would be my concern, thought, you know, the back-up if you have a positive result.' (GP ER49, pre-intervention, high prevalence) **'Any thoughts of using POCTs? If you were given them free?'** ' … Well, I wouldn’t feel particularly confident in the results there for me to deliver … And it’s not something I, kind of, trained, or would want to do … I think it’s an absolutely, it’s one of the biggest life-changing events, isn’t it? ... So it just feels outside of my depths to be honest.” (Nurse KTPN15K, pre-intervention, high prevalence) 'If you’re sending anyone for HIV, you would generally do a hepatitis as well … so if, if I’m going to do the point of care for HIV, I still have to send them for the hepatitis, so that doubles my work. So I would still send them for the blood test.' (GP KT8A, pre-intervention, high prevalence) '[Discussing POCTs] I think they’re fantastic. Yeah, because … half the problem is … like you do a pregnancy test, isn’t it? If you do pregnancy test for somebody, you say, "give me your urine and then I’ll see you in a week". You know, the anxiety that they are going through. Whereas, if you’ve got to deal with it there and then, as long as you’re doing it and the, you’ve done the build up to it. I think it’s a good thing.' (GP KTGP30J, pre-intervention, low prevalence)

Before the educational HIV workshop, GP staff generally only tested for HIV on patient’s request, or for patients that indicated that they may have a history of 'risky behaviour'. Some nurses implied that they already had a policy of offering HIV tests at sexual health checks, but could be more proactive with remembering to offer it consistently.

After the educational HIV workshop, there were still mixed perceptions of patients' acceptance of an HIV test, if it was offered routinely. Participants recognised that when patients were offered a full sexual health screen, they were more likely to accept a chlamydia test rather than an HIV test.

No participants referred to less invasive forms of specimen collection; all took venous blood specimens to send to laboratories for HIV testing. When asked, no practices, before or after the educational HIV workshop, were using point-of-care tests (POCTs).

Opinions of POCTs were generally negative, as it was not seen as logistically practical in a 10-minute consultation, especially if the test was positive; many participants felt they would be out of their depth giving positive results. A GP indicated that POCTs would be additional work, as, if it was clinically appropriate to test for HIV, they would always test for hepatitis as well, by taking blood. However, several sources could see the benefit of POCTs, as quicker results would mean less of a wait for the patient.

### Perceived behavioural control: the perceived ease or difficulty of routinely offering an HIV test, in line with NICE guidance; reflecting past experience as well as anticipated barriers (TPB) (Box 3)

**Box 3.** Quotes on perceived behavioural control: the perceived ease or difficulty of routinely offering an HIV test in line with NICE guidance; reflecting past experience as well as anticipated barriers (TPB)

'What to do with the results; what to advise the patient regarding the results; how it’s going to affect them in future life. Um, and also, I think there’s still more training required into the actual treatment, and so, once you’re starting to talk to somebody about having a screening test, they often, they want to know all the answers straightaway of, well, if I am positive, how, how is this going to be treated? How is it going to affect my life? And you’re also trying to put it into another consultation, um, it’s still a very scary thought for patients just to even have the test themselves, and so I think, just having a repeated discussion or training on the HIV side of it, just to keep our confidence going, would be beneficial.' (Nurse YNYKT125, post-intervention, low-prevalence) 'I think I would be quite nervous about it being a, used as a screening, and I would be concerned that the patient, the people that are doing the new patient health checks are probably the least experienced at being able to discuss patient concerns. It’s not the GPs that are discussing it and doing it, it’s the healthcare assistants, and I think there’s that, I think they would probably have concerns.' (Nurse YORA1, post-intervention, low prevalence)

The majority of participants were comfortable with taking a sexual history, but identified that further training would be needed, specifically on HIV clinical care pathways; improving confidence with regards giving a positive HIV result; and an update on the current treatments and outcomes.

Registering new patients and carrying out new patient checks is generally part of the duties of HCAs. There was concern that HCAs may need some additional training to ensure that they are comfortable with offering a routine HIV test, as they are often the least clinically experienced members of staff.

### Coherence: extent of understanding of purpose of educational HIV workshop, and clarity on role and responsibility of implementing guidance (NPT) (Box 4)

**Box 4.** Quotes on coherence: extent of understanding of purpose of educational HIV workshop and clarity on role implementing guidance (NPT)

'And they don’t need to have special counselling, as long as you have informed them that that’s what you’re testing for, and that you feel it’s appropriate for what they’ve come to you for, then that’s fine. But I think there still is that nervousness. But again, have I requested any HIV screening recently? Probably not.' (Nurse YORA1, post-intervention, low prevalence) 'But I remember learning an awful lot about HIV in a very short period of time that day. About the diseases that define it and who we should be testing. That’s what I got out of it. Because I see a lot of people with just slightly abnormal blood tests that I would never have thought of, but now I’m actually offering it to, for illnesses that I never even considered, that table of disease … defined illnesses [indicator illnesses]. That’s what came out of it for me.' (GP YORA4, post-intervention, low prevalence) '[Talking about their policy for HIV testing pre-intervention] I think some of the GPs offer HIV testing and some people would have sent patients to the local GUM [geniturinary medicine] clinic, and I think that’s maybe still the case. I think it’s probably not dealt with, not thought about enough really … I don't know, I suppose we learnt a bit on the training, but I don’t know if it’s changed a great deal.' (Nurse YNYCR122, post- intervention, low prevalence) '[when discussing indicator illnesses] Um yeah, that would be something that the GPs would pick up on and do forms for.' (Nurse LUCR121, post-intervention, high prevalence) 'They [doctor who had not attended the educational HIV workshop] keep twittering on about counselling ... only yesterday said to me, "oh and you have to request little special bottles and this for bloods", and I’m like "we don’t." And they don’t need to have special counselling, as long as you have informed them that that’s what you’re testing for, and that you feel it’s appropriate for what they’ve come to you for, then that’s fine.' (Nurse YORA1, post-intervention, low prevalence)

Participants thought that the educational workshop was useful and informative, especially updating knowledge on the policy around counselling, and the myths around declaring HIV tests to your insurance company. Several GPs indicated that the educational workshop had raised their awareness and that they tended to think more about HIV as a clinical diagnosis, especially for indicator illnesses, or for patients with non-specific or recurrent symptoms. Several mentioned being aware that there were missed opportunities for testing, and that having the list of indicator conditions would make it easier to offer a test.

Pre- and post-intervention, offering an HIV test to patients with indicator illnesses was considered the role of GPs, rather than nurses. Despite the educational workshop, some nurses reported that they would still probably refer to a GP within the practice, or to the GU clinic.

Current guidelines recommend that healthcare professionals should be able to obtain informed consent for an HIV test in the same way that they currently do for any other medical investigation.^22^ A few participants mentioned that other staff in the practice were unaware of the current policy around pre-counselling and consent, as they had not attended the educational workshop. This was a barrier as there was not a 'whole practice' approach.

### Collective action: work undertaken to implement the recommended NICE guidance (NPT) (Box 5)

**Box 5.** Quotes on collective action: work undertaken to implement the recommended NICE guidance (NPT)

'Well, actually, we’re all offering, if we do bloods, then we're offering them all saying, "it’s a routine procedure that we would offer the HIV testing as well."’ **'And is that just with routine blood tests? Are you offering them on any other, um, opportunities?'** 'Yeah, anyone that’s due, anyone that we do a blood form for basically, then we’re offering … I wouldn’t know percentage-wise what it is, but I think we’ve had quite a good uptake. Perhaps with the little bit of the financial element as well, that the tests are being offered more, so I think that’s probably spurred them on to some extent.' (Nurse LUCR121, post-intervention, high prevalence) **'So following the training, how did it change your practice?'** 'We have an alert now that pops up as well, which reminds us just to start the conversation … It’s with all new patients … Yeah, so anyone that registers gets that.' **'Is that an option that they have to tick or is that in a discussion that you have with ... '** 'We’ve trained our receptionists to say when they join. So when they fill out the forms the receptionists can say, "you’ll be offered a HIV testing, you can book in for bloods if you want to".' (GP YORA4, post-intervention, low prevalence) **'And the new patient check. Do you include HIV in that?'** 'Again, we try to, again, it’s something that, you know, isn’t always done, but yes, we try and offer that to all new patients who register at the surgery … there’s another enhanced service which is based on HIV screening for newly registered patients, so it may have been as part of that … I think the timing was good [referring to the HIV educational workshop], it came in at the same time it was all fitted in nicely with what was being run locally in our borough so it was, you know, it worked well … one reason why it became implemented in the, you know, because we’ve signed up to this, we have to, we need to do it.' (GP KICM124, post-intervention, high prevalence)

Some participants reported that they already had a policy of routinely offering an HIV test when taking blood for other tests. Some practices introduced the policy following the educational HIV workshop, and had been reminded to do this by a tick-box added to the blood request form. These participants indicated that there had been good uptake to this initiative by GP staff and patients.

After the educational HIV workshop, participants in the three practices in areas of high HIV prevalence had implemented the policy of offering all newly registered patients an HIV test. It was reported that this was facilitated by the coincidental timing of the HIV educational workshops with the introduction of a public health contract that gave payment for completed HIV tests.

### Cognitive participation: staff engagement in educational HIV workshop and general practice implementation (NPT) (Box 6)

**Box 6.** Quotes on cognitive participation: staff engagement in educational HIV workshop and general practice implementation (NPT)

'I think some of the doctors would think that the prevalence is so low that it’s not something they’re very motivated to screen for … You know, it would be much higher up on our agenda. The thing that makes HIV screening so difficult for us is that we are of such low prevalence that it’s really difficult to actually, you either, we either screen an awful lot of unnecessary worried well.' (GP NSCR121, post-intervention, low prevalence)

Although participants thought that the educational workshop was useful and informative, in areas of low HIV prevalence, they had not proactively offered HIV tests following the workshop. Reasons given for this included: low motivation, lack of funding, competing priorities, and feeling that it was unnecessary due to low prevalence in their areas.

After the educational HIV workshop, many GP staff, especially nurses, still found it difficult to offer an HIV test, due to a lack of knowledge, a lack of confidence, and overall nervousness or fear of dealing with a positive result.

### Reflexive monitoring: assessing impact of the work and making adjustments (NPT) (Box 7)

**Box 7.** Quotes on reflexive monitoring: assessing impact of the work and making adjustments (NPT)

**'Is it something that’s discussed between staff at all about testing?'** 'Initially when we had the training but I’d say we don’t really now.' (Nurse LUCR121, post-intervention, high prevalence) **'What other resources or support could have been given which might help to make more change happen?'** 'Probably if we had a meeting with the GPs and had a discussion about it and as to what the policy should be ... If the GPs were on board with offering it, then we would ... we’d probably do it ourselves more often as well ... I guess just regular updates to keep us up to date.' (Nurse YNYCR122, post-intervention, low prevalence)

Immediately following the educational workshop, some participants reflected that they had discussed how they would implement the NICE guidelines; however, these discussions had not been sustained enough to result in a change in practice.

## Discussion

### Summary

GP staff thought the HIV educational workshop was useful and informative, but in areas of low HIV prevalence, there was no reported increase in testing. GPs, specifically, reported that the workshops resulted in increased knowledge of and confidence in offering HIV tests based on indicator conditions, but participants still felt they needed additional HIV training around: clinical care pathways, to improve confidence offering tests; giving positive HIV results; and current treatments and outcomes. Participants did not see a place for POCTs in general practice.

Practices in areas of high HIV prevalence had implemented the offering of an HIV test to new patients. There was concern that HCAs, who undertake new patient appointments, may still need additional training to ensure that they are comfortable with offering a routine HIV test. Several practices had added the offer of an HIV test to their blood test forms and protocols, with reported good uptake by GP staff and patients.

### Strengths and limitations

This study addresses the recommendation from the 2016 NICE HIV testing guidelines^13^ that more research is needed on how to support healthcare professionals to offer HIV tests. It is timely and the findings can be used to inform future implementation of the guidance.

This qualitative approach provides more in-depth insights^23,24^ into the implementation of the NICE HIV testing guidelines in primary care than could be obtained by questionnaire.^25,26^ It increases understanding of how to optimise complex interventions to improve implementation on a larger scale, outside of research practices, with existing local authority-funded staff.^27–29^ However, it cannot be disregarded that the paucity of GPs from high prevalence areas may have had an impact on the findings of this study.

In the quantitative evaluation of the full 3Cs and HIV intervention,^8^ it was not possible to obtain routine GP practice HIV testing data to assess whether the educational HIV workshop had impacted on HIV testing rates. Therefore, qualitative findings are essential to reflect views and attitudes.^23,24,27,28^

The views of GP staff from areas of extremely high HIV prevalence (≥5 diagnosed per 1000 people) are not reflected, as this definition was added to the 2016 NICE guidelines, after this study. No GP staff interviewed had used POCTs for HIV testing, so discussions were centred on perceptions, rather than actual experiences of POCTs. However, the sample interviewed reflects the views of a range of GP staff from a variety of settings, based on geographical location, HIV prevalence, size of practice, and population characteristics.

### Comparison with existing literature

A previous systematic review of UK-based interventions to increase HIV testing^30^ supported education of healthcare practitioners on HIV clinical indicator diseases, HIV risk factors, proactive testing, and use of opt-out approaches, particularly in practices in high prevalence areas.^31^ HIV testing rates in primary care will also be dependent on patient acceptability. A study in Cornwall, UK found that 97% (328/339) of patients would be willing to have an HIV test if presenting with a medical problem known to be associated with HIV.^32^ A London primary care centre attained a 75.4% (1002/1329) uptake when patients were offered an HIV test.^33^ Qualitative work in primary care shows that patients display a clear preference for HIV testing to be offered at the GP practice over other sexual health service providers.^34^ Although the literature indicates that patients would be open to an offer of HIV testing in general practice,^35–37^ this current qualitative evaluation suggests that, despite an educational HIV workshop, GP staff still lack confidence in and experience of offering HIV tests routinely in consultations. This would have to be addressed before considering rolling out routine opportunistic testing in all consultations, as encouraged in the 2016 HIV guidelines for areas of extremely high HIV prevalence.^13^

### Implications for practice

The findings of this qualitative study indicate that implementation of current NICE HIV testing guidelines^13^ will need more support than just a single workshop, especially to implement testing guidelines for indicator conditions in areas of low HIV prevalence. Further workshops or additional training should include very clear care pathways for dealing with and communicating with HIV positive patients, as participants suggested that they would be more inclined to offer the test if they were comfortable and confident delivering a positive result. Additional role-play or discussions around the scripts used in the educational workshop, suggesting how to offer an HIV test, may help participants feel more confident and facilitate testing with new patient checks, routine bloods, cervical smears, and other sexual health checks. HCAs may need specific training to ensure that they are skilled in offering HIV testing within new patient checks or blood test requests.

Local authorities could incorporate public health contracts for HIV testing simultaneously with educational training, as GP staff reported that this incentive facilitated routine offers of HIV tests to all new patients. As GP staff did not think that POCT was feasible in a routine appointment, these tests may be better placed in specialist sexual health settings, as suggested in the NICE guidelines.

## References

[1] Kirwan P, Chau C, Brown A (2016). HIV in the UK: 2016 report.

[2] Marks G, Crepaz N, Janssen RS (2006). Estimating sexual transmission of HIV from persons aware and unaware that they are infected with the virus in the USA. AIDS.

[3] Nakagawa F, Lodwick RK, Smith CJ (2012). Projected life expectancy of people with HIV according to timing of diagnosis. AIDS.

[4] Sabin C (2012). Review of life expectancy in people with HIV in settings with optimal ART access: what we know and what we don't. J Int AIDS Soc.

[5] The National Institute for Health and Care Excellence (2011). *HIV testing: increasing the uptake of HIV testing among men who have sex with men* [Public health guideline: PH34].

[6] The National Institute for Health and Care Excellence (2011). *Increasing the uptake of HIV testing among Black Africans in England* [Public health guideline: PH33].

[7] Town K, McNulty CA, Ricketts EJ (2016). Service evaluation of an educational intervention to improve sexual health services in primary care implemented using a step-wedge design: analysis of chlamydia testing and diagnosis rate changes. BMC Public Health.

[8] Town K, Ricketts EJ, Hartney T (2015). Supporting general practices to provide sexual and reproductive health services: protocol for the 3Cs & HIV programme. Public Health.

[9] Allison R, Lecky DM, Town K (2017). Exploring why a complex intervention piloted in general practices did not result in an increase in chlamydia screening and diagnosis: a qualitative evaluation using the fidelity of implementation model. BMC Fam Pract.

[10] Ajzen I (2018). Theory of planned behaviour. http://people.umass.edu/aizen/index.html.

[11] Ajzen I (2002). Perceived behavioral control, self-efficacy, locus of control, and the theory of planned behavior. J Appl Soc Psychol.

[12] HIV in Europe Initiative (2015). HIV indicator conditions: guidance for implementing HIV testing in adults in health care settings. http://hiveurope.eu/Portals/0/Guidance.pdf.

[13] National Institute for Health and Care Excellence (NICE), Public Health England (PHE) (2016). HIV testing: increasing uptake among people who may have undiagnosed HIV (joint NICE and Public Health England guideline). https://www.nice.org.uk/guidance/ng60/resources/hiv-testing-increasing-uptake-among-people-who-may-have-undiagnosed-hiv-pdf-1837567043269.

[14] McNulty CA, Hogan AH, Ricketts EJ (2014). Increasing chlamydia screening tests in general practice: a modified Zelen prospective cluster randomised controlled trial evaluating a complex intervention based on the theory of planned behaviour. Sex Transm Infect.

[15] Guest G, Bunce A, Johnson L (2006). How many interviews are enough? An experiment with data saturation and variability. Field methods.

[16] Patton MQ (2005). Qualitative research. Encyclopedia of Statistics in Behavioral Science.

[17] Newton N (2010). The use of semi-structured interviews in qualitative research: strengths and weaknesses. Exploring Qualitative Methods.

[18] May CR, Mair F, Finch T (2009). Development of a theory of implementation and integration: normalization process theory. Implement Sci.

[19] Charmaz K (2014). Constructing grounded theory.

[20] Gale NK, Heath G, Cameron E (2013). Using the framework method for the analysis of qualitative data in multi-disciplinary health research. BMC Med Res Methodol.

[21] de Brún T, O'Reilly-de Brún M, O'Donnell CA (2016). Learning from doing: the case for combining normalisation process theory and participatory learning and action research methodology for primary healthcare implementation research. BMC Health Serv Res.

[22] Knott L (2015). HIV Counselling. https://patient.info/doctor/hiv-counselling#nav-5.

[23] Smeets HM, Kuyvenhoven MM, Akkerman AE (2009). Intervention with educational outreach at large scale to reduce antibiotics for respiratory tract infections: a controlled before and after study. Fam Pract.

[24] Cabana MD, Rand CS, Powe NR (1999). Why don't physicians follow clinical practice guidelines? A framework for improvement. JAMA.

[25] Curry LA, Nembhard IM, Bradley EH (2009). Qualitative and mixed methods provide unique contributions to outcomes research. Circulation.

[26] Creswell JW, Clark VLP (2007). Designing and conducting mixed methods research.

[27] Pope C, Mays N, Popay J (2007). Synthesising qualitative and quantitative health evidence: a guide to methods..

[28] Greenhalgh T, Annandale E, Ashcroft R (2016). An open letter to *The BMJ* editors on qualitative research. BMJ.

[29] Thornton AC, Rayment M, Elam G, HINTS Study Group (2012). Exploring staff attitudes to routine HIV testing in non-traditional settings: a qualitative study in four healthcare facilities. Sex Trans Infect.

[30] Davies C, Gompels M, May M (2015). Public and healthcare practitioner attitudes towards HIV testing: review of evidence from the United Kingdom (UK). Int STD Res Rev.

[31] Howland C, Majewska W (2013). Training resource designed to increase frequency of HIV testing in non-GUM settings displays promising outcomes. HIV Med.

[32] Drayton R, Keane F, Prentice E (2010). Patients' attitudes towards increasing the offer of HIV testing in primary and secondary care. Int J STD AIDS.

[33] Rayment M, Thornton A, Mandalia S, HINTS Study Group (2012). HIV testing in non-traditional settings — the HINTS study: a multi-centre observational study of feasibility and acceptability. PLoS ONE.

[34] Jones LF, Ricketts E, Town K (2017). Chlamydia and HIV testing, contraception advice, and free condoms offered in general practice: a qualitative interview study of young adults' perceptions of this initiative. Br J Gen Pract.

[35] Fraisse T, Fourcade C, Brazes-Sanz J (2016). A cross sectional survey of the barriers for implementing rapid HIV testing among French general practitioners. Int J STD AIDS.

[36] Hindocha S, Charlton T, Rayment M (2013). Feasibility and acceptability of routine human immunodeficiency virus testing in general practice: your views. Prim Health Care Res Dev.

[37] Glew S, Pollard A, Hughes L (2014). Public attitudes towards opt-out testing for HIV in primary care: a qualitative study. Br J Gen Pract.

